# Association between incidental dose outside the prostate and tumor control after modern image-guided radiotherapy

**DOI:** 10.1016/j.phro.2020.12.003

**Published:** 2021-01-05

**Authors:** Marnix Witte, Floris Pos, Luca Incrocci, Wilma Heemsbergen

**Affiliations:** a Department of Radiation Oncology, The Netherlands Cancer Institute, Amsterdam, The Netherlands; b Department of Radiation Oncology, Erasmus Medical Center, Rotterdam, The Netherlands

**Keywords:** Radiotherapy, Prostate cancer, Freedom from failure, Incidental dose, Dose modelling

## Abstract

**Background and purpose:**

External beam radiotherapy for prostate cancer deposits incidental dose to a region surrounding the target volume. Previously, an association was identified between tumor control and incidental dose for patients treated with conventional radiotherapy. We investigated whether such an association exists for patients treated using intensity modulated radiotherapy (IMRT) and tighter margins.

**Materials and methods:**

Computed tomography scans and three-dimensional treatment planning dose distributions were available from the Dutch randomized HYPRO trial for 397 patients in the standard fractionation arm (39 × 2 Gy) and 407 patients in the hypofractionation arm (19 × 3.4 Gy), mainly delivered using online image-guided IMRT. Endpoint was any treatment failure within 5 years. A mapping of 3D dose distributions between anatomies was performed based on distance to the surface of the prostate delineation. Mean mapped dose distributions were computed for patient groups with and without failure, obtaining dose difference distributions. Random patient permutations were performed to derive p values and to identify relevant regions.

**Results:**

For high-risk patients treated in the conventional arm, higher incidental dose was significantly associated with a higher probability of tumor control in both univariate and multivariate analysis. The locations of the excess dose mainly overlapped with the position of obturator internus muscles at about 2.5 cm from the prostate surface. No such relationship could be established for intermediate-risk patients.

**Conclusions:**

An association was established between reduced treatment failure and the delivery of incidental dose outside the prostate for high-risk patients treated using conventionally fractionated IMRT.

## 1 Introduction

Prostate cancer is the second most frequently diagnosed cancer [1] and the sixth leading cause of cancer death in males worldwide [2]. Radiotherapy (RT) is a viable option for clinically localized prostate cancer [3], and can optionally target regions suspected of subclinical involvement with reduced dose. There might be a rationale to target elective regions around the prostate and around pelvic lymph nodes, since several studies have shown considerable rates of extra-prostatic disease in prostatectomy series, and lymph node involvement in lymph node dissection series and imaging studies. However, the clinical benefit of such elective fields in the primary RT setting is questioned since previous randomized trials in the three-dimensional conventional radiotherapy (3DCRT) era did not show any benefit of adding such fields [4], [5] while at the same time these are associated with increased toxicity rates.

With external beam radiotherapy, incidental dose is unavoidably delivered outside the target volume, potentially targeting subclinical disease. While improved diagnostic magnetic resonance imaging (MRI) and lymph node staging using prostate-specific membrane antigen (PSMA) positron emission tomography (PET) [6] may increase the detection rate of organ non-confined disease (and thus lead to a stage migration), no imaging technique is perfect and those patients clinically diagnosed with localized disease may yet suffer undetectable loco-regional progression. Previously a relationship was demonstrated in a prostate cancer patient trial population treated with 3DCRT, where dose delivery in the obturator region was significantly associated with freedom from failure [7]. A prophylactic incidental dose-response effect was confirmed in a randomized toxicity trial [8] where tumor control for high-risk patients was found to be superior for rectangular fields compared to 3DCRT.

Nowadays patients are treated with high dose conformity and small margins using image guided intensity modulated radiotherapy (IG-IMRT), and the dose at major lymph node stations is for all prostate cancer patients below clinically effective levels. With IG-IMRT, dose levels in excess of 50 Gy are typically only present within the first 3 cm from the surface of the delineated prostate. In the current study, we investigated whether a relationship between incidental dose and tumor control can be found in an IG-IMRT setting. To this purpose, we performed a retrospective unplanned analysis of both study arms of the prospective HYPRO study [12] in which patients were randomized between standard fractionation and hypofractionation.

## 2 Materials and methods

### 2.1 Patients

In the Dutch multi-center open-label randomized phase III trial ‘HYPRO’, patients with histologically confirmed intermediate or high-risk localized prostate cancer were recruited and randomly allocated to either conventionally fractionated (39 × 2*.*0 Gy, CF) or hypo-fractionated (19 × 3*.*4 Gy, HF) radiotherapy. The HYPRO trial was approved by the Medical Ethics Committee of the Erasmus Medical Center in Rotterdam, the Netherlands (06-045). The participants provided their written informed consent to participate in this study. The study population and procedures have previously been described in detail [12]. Based on the estimated risk of seminal vesicle (SV) involvement [13], [14], three treatment groups were defined for SV dose prescription. For patients in group 1 (risk of SV involvement *<* 10%) the clinical target volume was the delineated prostate only, in groups 2 and 3 the SV were part of the clinical target volume (CTV). In group 3 (risk of SV involvement *>* 25%) these were prescribed the same dose as the prostate, but in group 2 the SV prescription was lowered to 70 Gy and 54*.*5 Gy for CF and HF, respectively. Treatment planning CT scans with delineated prostate and 3D dose distributions as exported from the treatment planning system were available.

Since a potential effect of extra-prostatic dose would be expected to correlate with the risk of organ non-confined disease, patients were subdivided in intermediate and high-risk subgroups. Treatment failure was defined as biochemical relapse [15], clinical relapse, loco-regional or distant relapse, or start of hormone therapy, whichever occurred first. All failures within 5 years were labeled as ‘failure/event’ for the current analyses of incidental dose correlations.

For our analysis we considered patients with sufficient follow-up from the updated database [16] and available treatment planning CT and 3D dose distribution, at intermediate (CF: 90 patients, HF: 86 patients) and high prognostic risk (CF: 249 patients, HF: 241 patients), analyzing in total 666 out of the 804 patients (83%) of the total HYPRO study population previously available for relapse-free survival analysis. Relapse was defined as biochemical relapse, clinical relapse, locoregional or distant relapse, or start with hormonal therapy, whichever happened first. Biochemical relapse was defined as prostate-specific antigen (PSA) concentration greater than the present nadir plus 2 ng/mL, without backdating. Patients who died without evidence of previous relapse and not because of toxic effects of treatment were censored at date of death.

### 2.2 Dose mapping

A dose mapping procedure was followed [7] based on the prostate delineation as it was used for treatment planning, and the 3D dose distribution exported form the treatment planning system. Outside the prostate, two points in the anatomies of different patients map to the same location if they have the same direction with respect to the center of mass of the prostate, and the same distance to the triangulated prostate surface. Inside the prostate delineation, the relative distance between center of mass and surface should correspond. To aggregate results and enable anatomical interpretation, the Visible Human data set [17] was used as anatomical template with a prostate delineation that was specifically generated to resemble a radiotherapy CTV, but yet to have a smooth surface, minimizing potential mapping artifacts. A sufficiently large dose grid with 4 mm cubic voxels was constructed around the prostate. For a given patient the dose values at these grid locations were computed by trilinear interpolation at the corresponding mapped location in the patient’s 3D planning dose distribution. Dose differences by failure were computed by first evaluating the average 3D mapped dose distribution over the patient subgroup who had experienced treatment failure, minus the average dose over the failure-free patient subgroup. In-house developed software ‘Match42′ was used, consisting of C^++^ computational modules, a GUI built in Delphi, and a Python scripting layer.

### 2.3 Statistical analysis

To test for significantly different mean dose differences between outcome subgroups, a permutation method was used [18]. Patient permutations were performed, randomly relabeling patients between the failure and non-failure cohorts. For each permutation a map of dose differences was generated. Dose differences were collected for those voxels which on average (over both groups) received at least 10% of the prescribed dose, and the 99th percentile maximum in absolute value was recorded (i.e. the maximum absolute difference after removing the 1% voxels with highest absolute difference). Repeating these permutations 10,000 times, a cumulative histogram was constructed. For an observed dose difference map a global p-value was computed as the fraction of randomizations with a 99th percentile maximum dose difference higher than observed.

Furthermore, patient permutations were performed to estimate q-value maps which could be thresholded to identify subsets of voxels at given estimated false discovery rate [19]. For each permutation a map of p-values was generated using a *t*-test per voxel, and a global null histogram of p-values was accumulated over the permutations. From these, the proportion of true null hypotheses for a given p-value cut-off was estimated [20], and used to translate the map of p-values for an observed dose difference to the corresponding q-value map. The estimated proportion of falsely discovered voxels is expected to be conservative and robust against positive correlations between voxels (see [21] and references therein).

For maps with a significant difference at the permutation test (*p <* 0*.*05), a location was selected from a region with low q-values, and the dose for each patient was extracted. Using dose cut-offs chosen to be relevant for the observed values, Kaplan-Meier survival analyses were performed (IBM Corp. Released 2017. IBM SPSS Statistics for Windows, Version 25.0. Armonk, NY: IBM Corp.). Also, these mapped dose values were added to multivariate Cox regression models which were constructed from established risk factors for treatment failure (T-stage, PSA, Gleason, adjuvant hormone therapy) which were significant at univariate analysis.

### 2.4 Linear-quadratic dose correction

At intermediate dose levels outside the target area the daily dose levels are lower than inside the target, therefore non-linear biological effects depend not only on the fractionation scheme, but also on location. To allow comparison of these effects we derived mapped dose distributions biologically equivalent to 2 Gy using the linear quadratic model assuming *α/β* ratios of 3 Gy and 1 Gy, and computed maps of mean dose and standard deviation of dose over the CF and HF patient groups.

## 3 Results

### 3.1 Dose difference maps

**Fig. 1:**
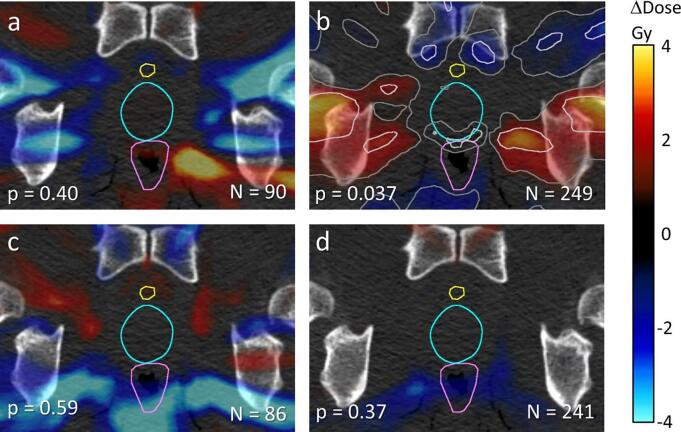
Dose differences by failure mapped around the prostate in axial view with delineated structures bladder (yellow), prostate (cyan) and rectum (magenta). Light and dark gray lines indicate q-values of 25% and 50%, respectively. Global p-values were based on random patient permutations. **1a** Intermediate-risk CF; **1b** high-risk CF; **1c** intermediate-risk HF; **1d** high-risk HF. (For interpretation of the references to colour in this figure legend, the reader is referred to the web version of this article.)

**Fig. 2:**
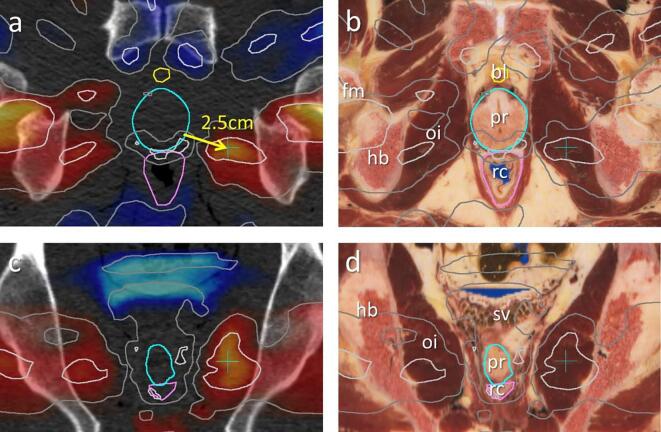
Axial (top) and coronal (bottom) slices through the CT set (left) and the anatomy based on cryo slices (right) of the Visible Human data set with delineated structures bladder (yellow), prostate (cyan) and rectum (magenta). Light and dark gray lines indicating q-values of 25% and 50% correspond to the results in Fig. 1b for high risk CF patients. Dose values at the location of the cyan cross at 2*.*5 cm from the delineated prostate were used for subsequent survival analysis and logistic regression. Abbreviations: bl: bladder, fm: femur, hb: hip bone, oi: obturator internus, pr: prostate, rc: rectum, sv: seminal vesicles. (For interpretation of the references to colour in this figure legend, the reader is referred to the web version of this article.)

Dose difference maps around the prostate (i.e. average dose map of subgroup ‘no failure’ within 5 years minus average dose map of subgroup ‘failure’), were generated for the two risk groups and separately for HF and CF (Fig. 1). In the intermediate-risk group positive and negative dose differences up to 10 Gy were observed in both the CF and HF arms, however the numbers of patients in these groups were relatively small (N = 90 and N = 86, respectively), and these differences were not significant in the permutation tests. For the larger group of high-risk patients, only smaller dose differences were observed for the HF group (241 patients), which were not significant. Only for the CF arm the *p* derived from permutation (*p* = 0*.*037) was significant at the 5% level, and for this group mainly positive dose differences at *q*-values less than 25%, i.e. with an expected proportion of false discoveries below 25%, were observed around the prostate in a region overlapping with obturator internus muscles (Fig. 2). Most prominent differences occurred at about 2*.*5 cm distance from the prostate surface where typically incidental dose levels of 40 Gy to 60 Gy can be found in the CF treatment plans.

### 3.2 Adjusting for time-to-event and other variables

**Fig. 3:**
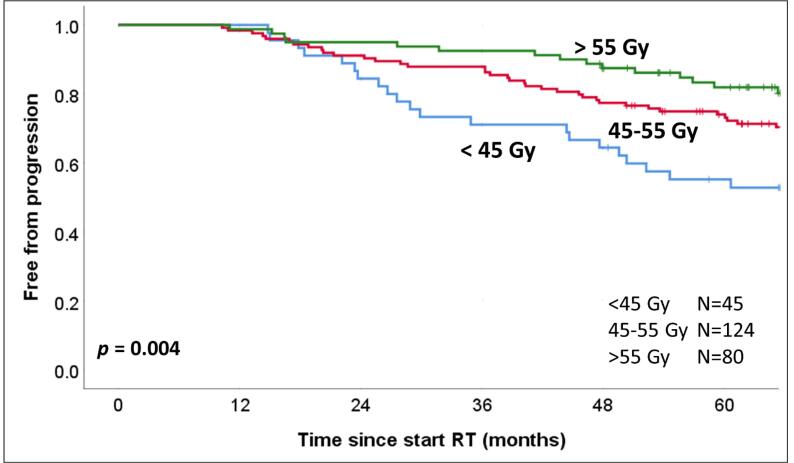
Kaplan Meier curves (free from tumor progression) for high risk patients in the conventional treatment arm, using 45 Gy and 55 Gy dose cut-off levels in the location marked with a cyan cross in Fig. 2. (For interpretation of the references to colour in this figure legend, the reader is referred to the web version of this article.)

Significantly different freedom from tumor progression (*p <* 0*.*01) was observed for the high-risk CF group using dose cutoffs of 45 Gy and 55 Gy (Fig. 3). Table 1 shows results for univariate (UV) and multivariate (MV) analysis. As expected, Gleason score and AHT had an impact both for CF and HF patients. When adding the extra-prostatic point dose values as a variable in the CF group in a time-to-event Cox regression, it had significant predictive power both at univariate (HR = 0*.*61, *p <* 0*.*01) and multivariate analysis (HR = 0*.*57, *p <* 0*.*01), indicating that the HR was stable and there was no confounding effect of other clinical variables in the dose maps.

**Table 1:** Cox regression based on relapse free survival at 5 yr from randomization for high risk patients in the two treatment arms. Univariately (borderline) significant clinical covariates were tested in multivariate models, and for the conventional patients the recorded extraprostatic dose values were optionally added.

CF N = 249	UV		MV		MV	
variable	HR	*p*	HR (95% CI)	*p*	HR (95% CI)	*p*
T-stage (3 vs. 1 2)	0.70	0.13				
Gleason (≥vs. *<* 8)	1.64	0.031	1.86 (1.2 3.0)	0.009	2.05 (1.3 3.3)	0.003
PSA (*>*vs. ≤ 20ngml^−1^)	1.20	0.4				
AHT (yes vs. no)	0.58	0.034	0.50 (0.3 1.0)	0.009	0.43 (0.3 0.7)	0.002
Hospital (ref = largest)		0.3				
D^+^ (per 10 Gy)	0.61	0.001			0.57 (0.4 0.8)	*<*0.001

HF N=241	UV		MV	
variable	HR	*p*	HR (95% CI)	*p*
T-stage (3 vs. 1 2)	0.81	0.4		
Gleason (≥vs.*<* 8)	2.22	0.001	2.24 (1.4 3.6)	0.001
PSA (*>*vs. ≤20ngml^−1^)	0.94	0.8		
AHT (yes vs. no)	0.61	0.065	0.60 (0.4 1.0)	0.058
Hospital (ref = largest)		0.9		

### 3.3 Fractionation effects

**Fig. 4:**
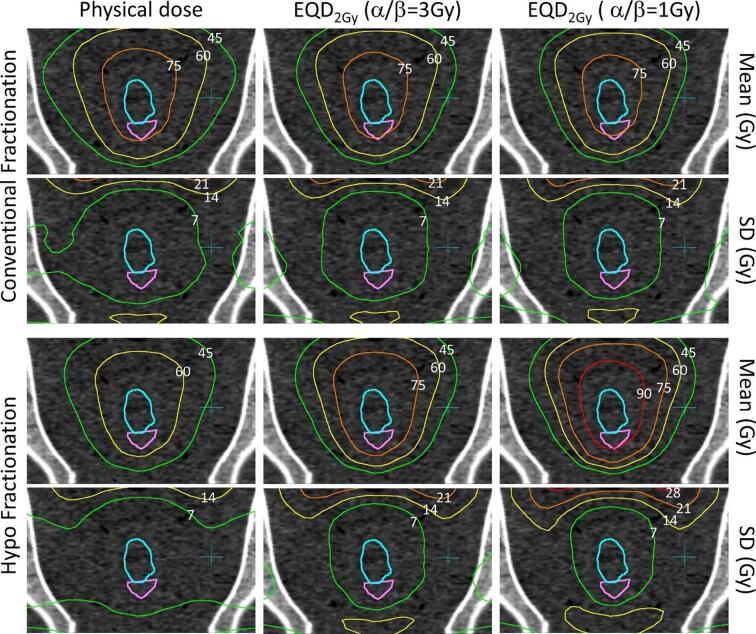
Mean and standard deviation of dose distributions mapped around the prostate in coronal view for high-risk patients treated with conventional fractionation (top rows) and hypofractionation (bottom rows), using physical dose and EQD_2Gy_ with *α/β* = 3 Gy and *α/β* = 1 Gy (columns left to right). Delineated structures are prostate (cyan) and rectum (magenta). (For interpretation of the references to colour in this figure legend, the reader is referred to the web version of this article.)

For CF the high dose region was largely unaffected by fractionation correction, while the biologically corrected intermediate dose ranges became concentrated closer to the target as the relative contribution of the quadratic term in the linear-quadratic model was increased (Fig. 4). At the same time, standard deviation of the biologically corrected dose was somewhat increased. For the HF group the physical dose to the target was lower at 64 Gy, but became comparable to the CF arm after EQD_2Gy_ correction with *α/β* = 3 Gy and superior both in- and outside the target when using *α/β* = 1 Gy. The same was observed for the standard deviation of the dose.

## 4 Discussion

An association was found between incidental dose around the prostate and tumor control probability for high-risk patients treated with conventionally fractionated modern RT techniques, with a benefit for higher extra-prostatic dose levels. The critical incidental dose levels in this region were typically in the range of 40 Gy to 60 Gy, comparable to broadly recognized elective dose levels of 46 Gy to 50 Gy appropriate to target subclinical disease.

To our knowledge no other reports of a correlation between tumor control and extra-prostatic dose for IMRT patients have been published. The reporting of increased failure rates in an IMRT trial [11] with intensive image guidance and reduced margins could be interpreted as a hint towards such an effect. An alternative interpretation could be that residual geometric uncertainties were underestimated.

Treatment effects of unintentional radiation dose have been considered in other tumor sites. A recent study [9] demonstrated that for bladder cancer patients treated with conformal radiotherapy, high levels of incidental dose were delivered mainly to obturatorial lymph nodes, and these authors hypothesized this may have caused a lack of clear benefit in trials testing nodal irradiation. Another study [10] investigated incidental dose being prophylactic for nodal relapse in lung cancer patients. In such cases however, disease stage and risk of nodal involvement may be quite different from those of a prostate radiotherapy population.

A main concern with the retrospective analysis of imaging data are the multiple testing issues introduced by the large numbers of voxels being analyzed. By extensively using permutation approaches we were able to derive a single p-value for a dose difference map based on the observed maximum difference, and we converted less-informative per-voxel p-values to q-values which allow a quantitative interpretation. This way we were able to show that observed dose differences were not likely purely coincidental, and we could identify the anatomical regions where dose differences were most prominent. However, dose values along an external beam direction are dependent, and patterns of variation are correlated due to symmetries in optimized radiation plans. It is therefore not possible to identify isolated anatomical effect regions; indeed, the location that was selected for point dose analysis should only be considered indicative for a possible effect in relevant regions.

A significant result was only found for dose differences in the high-risk patient group in the conventional arm. For the smaller groups of intermediate risk patients, the observed dose differences were of comparable magnitude, however as the permutation tests indicated these could well be the result of stochastic variation; only for a sufficiently large sample of patients a stable average dose distribution is to be expected.

An association was not found for the subgroup receiving hypo-fractionated treatment. From this observation, two distinct implications can be inferred. Firstly, the non-observation in the HF arm strengthens the notion of a causal relation in the CF arm between the observed dose differences and treatment effect, i.e. the existence of an extra-prostatic dose-effect relation. This follows from consideration of the opposite, so assuming some unidentified clinical factor correlating both with extra-prostatic dose and with outcome. As the CF and HF patients were subdivided purely by randomization, various patient and treatment characteristics are expected to be evenly distributed; indeed, this is the rationale to perform a randomization. So, if such a factor would indirectly have led to the observed dose differences in the CF arm, then this factor should be expected to also have led to similar dose differences in the HF arm. In other words, the reason dose differences were observable should be found in the difference between the randomization arms, which are different only in the way patients received radiation dose. Secondly, the linear-quadratic model with *α/β* = 3 Gy seems inappropriate to describe the mechanism that caused the observed dose differences. Since the EQD_2Gy_ corrected CF and HF dose distributions at *α/β* = 3 Gy are highly similar, an extra-prostatic dose effect visible in one arm would also be expected to manifest in the other arm. This suggests the extra-prostatic dose effect requires a much lower *α/β* ratio, or perhaps another model altogether (e.g. involving repair kinetics, or daily dose level thresholds).

As previously described for this data set [16], high-risk patients (Gleason 8) in the HF arm experienced significantly fewer local recurrences than in the CF arm, even though there was no difference in overall failure free survival. This suggests that for a high-risk subgroup the HF scheme may be more effective to treat the primary tumor (receiving full dose prescription), but at the same time perhaps less effective at eradicating subclinical disease receiving intermediate levels of incidental dose.

The main dose differences were found to overlap with the obturator internus muscles at a few centimeters away from the prostate, which is where the external beams have their field edges and therefore the largest dose gradients occur. Due to the strong spatial correlations in the dose distributions of external beams, the location of a large dose variation does not necessarily pin-point the location of an underlying mechanism. The identified regions were roughly consistent with previous findings [7], however these analyses concerned patients treated with 3DCRT techniques and larger margins, for which critical dose differences were located somewhat further away from the prostate. For the case where rectangular fields had superior tumor control compared to 3DCRT [8] the involved regions were located yet further away from the prostate.

Our retrospective image analysis cannot prove the nature of a potential underlying cause for the observed dose differences, however various observations may guide its hypothesis. The dose difference regions seem to be too far away for extracapsular extensions as known from surgical series, which is usually observed to extend only a few millimeters outside the prostatic capsule, whereas radiotherapy prostate CTV delineations (on which the dose mapping was based) even tend to be rather wide around the actual organ to accommodate delineation uncertainties. Identified dose differences do extend towards the caudal part of the obturatorial lymph node region, however this region extends cranially on an ascending pathway toward the retroperitoneum [22] outside the reach of the radiation fields. It seems unlikely that occult lymph node metastasis only affected the most proximal stations for a large part of our study population [23]. Our results were evaluated at five years from randomization, and while this is a common timeframe for the primary endpoint of a clinical trial, development of the disease may span a much longer time. Lymph node metastases as may be imaged at treatment failure using PSMA PET-CT may occur years after RT, at which time the patient usually presents with many distant lesions simultaneously [24]. Interestingly, these authors also report a surprisingly large fraction of patients (up to 15%) with lymph node recurrences in the mesorectum. This indicates that the disease must have had ample time to spread before leading to a PSA rise, and that this process would often involve the prostate’s local surroundings. This leaves open the question where the tumor cells that led to metastasis were located at the time of irradiation. While recurrent prostate carcinoma after RT is often found at the site of the primary tumor [25], [26], this does not necessarily mean that distant metastases must have been seeded by a such local recurrence. Possibly, locally spread out tumor outside the high dose region could survive RT with less radiation damage than the cells receiving full dose inside the tumor, and despite lower numbers be quicker to metastasize; more so because spread out cells would constitute a sub-population of tumor cells with a proven ability to invade surrounding tissues. Increased incidental dose levels to such regions could then lead to a delay in the process, resulting in a treatment failure difference after five years; it is not known whether these differences would remain significant at longer follow-up. The existence of isolated deposits (‘islets’) of tumor cells at a distance from the primary tumor has been studied in e.g. breast cancer [27] and NSCLC [28], however the spread of tumors within such organs may not easily translate to the spread of prostate carcinoma in the pelvis. In particular, the fact that dose difference regions mainly overlapped with obturator muscles complicates such an interpretation, as muscular tissues can resist a direct metastatic invasion quite well [29]. Considering that the main route for distant metastasis appears to be through blood vessels [30], perhaps invasion of the muscular vasculature plays a role, e.g. through the formation of intra- or perivascular tumor micro-environments seeded by (clusters of) cancer cells which intravasate at the site of the primary tumor. Such micro-environments could aid in the further adaptation of cancer cells to acquire the traits necessary for successful extravasation at a distant site, and subsequent colony formation. The existence of such micro-environments outside the primary tumor site has however never been demonstrated.

The results for the high-risk CF patients show positive dose differences at low *q*-values away from the prostate, but also regions of low *q* at the interface between prostate and rectum, for which dose differences are small but negative (see Fig. 1). In this region there is very little dose variation, which means that a given (small) dose difference easily attains a low *q*-value. In IMRT optimization an improved target conformity comes at the cost of increased complexity, and to reduce dose in one place it is generally necessary to allow dose increase elsewhere. Likely this is the cause of the observed cold region: plans with high PTV conformity towards the rectum required increased monitor units in the lateral beams which could then lead to the observed effects.

As the underlying cause of observed dose differences is uncertain, we cannot currently advise to change the RT target for high risk patients in any particular way. However, based on current and previous findings we would suggest to incorporate dose delivery around the primary target when analyzing trial results, as such interactions going unnoticed may skew the analysis and obfuscate actual study results. If more studies can be shown to harbor similar effects, the combined body of evidence may help to formulate a sound hypothesis for a causal relation, and eventually enable prospective research to improve RT for these patients.

The HYPRO data suggest an association between treatment failure and the incidental delivery of dose outside the prostate for high risk patients using conventional fractionation. The locations of identified dose differences may indicate local spread of subclinical disease in relevant tissues close to the prostate.

## Declaration of Competing Interest

The authors declare that they have no known competing financial interests or personal relationships that could have appeared to influence the work reported in this paper.
